# Low flow extracorporeal CO_2_ removal in ARDS patients: a prospective short-term crossover pilot study

**DOI:** 10.1186/s12871-017-0445-9

**Published:** 2017-11-28

**Authors:** Harlinde Peperstraete, Sunny Eloot, Pieter Depuydt, Filip De Somer, Carl Roosens, Eric Hoste

**Affiliations:** 1 Intensive Care Unit, Ghent University Hospital, De Pintelaan 185, 9000 Ghent, Belgium; 2 Renal Division, Ghent University Hospital, De Pintelaan 185, 9000 Ghent, Belgium; 3 Ghent University, Ghent, Belgium; 4 Department of Cardiac Surgery, Ghent University Hospital, De Pintelaan 185, 9000 Ghent, Belgium; 5 Research Foundation-Flanders (FWO), Brussels, Belgium

**Keywords:** Acute respiratory distress syndrome, Lung protective mechanical ventilation, Extracorporeal carbon dioxide removal, Plateau pressure, Driving pressure

## Abstract

**Background:**

Lung protective mechanical ventilation (MV) is the corner stone of therapy for ARDS. However, its use may be limited by respiratory acidosis.

This study explored feasibility of, effectiveness and safety of low flow extracorporeal CO_2_ removal (ECCO_2_R).

**Methods:**

This was a prospective pilot study, using the Abylcap® (Bellco) ECCO_2_R, with crossover off-on-off design (2-h blocks) under stable MV settings, and follow up till end of ECCO_2_R. Primary endpoint for effectiveness was a 20% reduction of PaCO_2_ after the first 2-h. Adverse events (AE) were recorded prospectively.

We included 10 ARDS patients on MV, with PaO_2_/FiO_2_ < 150 mmHg, tidal volume ≤ 8 mL/kg with positive end-expiratory pressure ≥ 5 cmH_2_O, FiO_2_ titrated to SaO_2_ 88–95%, plateau pressure ≥ 28 cmH_2_O, and respiratory acidosis (pH <7.25).

**Results:**

After 2-h of ECCO_2_R, 6 patients had a ≥ 20% decrease in PaCO_2_ (60%); PaCO_2_ decreased 28.4% (from 58.4 to 48.7 mmHg, *p* = 0.005), and pH increased (1.59%, p = 0.005). ECCO_2_R was hemodynamically well tolerated. During the whole period of ECCO_2_R, 6 patients had an AE (60%); bleeding occurred in 5 patients (50%) and circuit thrombosis in 3 patients (30%), these were judged not to be life threatening.

**Conclusions:**

In ARDS patients, low flow ECCO_2_R significantly reduced PaCO_2_ after 2 h, Follow up during the entire ECCO_2_R period revealed a high incidence of bleeding and circuit thrombosis.

**Trial registration:**

https://clinicaltrials.gov identifier: NCT01911533, registered 23 July 2013.

**Electronic supplementary material:**

The online version of this article (10.1186/s12871-017-0445-9) contains supplementary material, which is available to authorized users.

## Background

Acute Respiratory Distress Syndrome (ARDS) is a frequently occurring disorder in critically ill patients, and is associated with poor short-term and long-term outcomes [1, 2]. Lung protective mechanical ventilation (MV), i.e. use of a tidal volume (V_T_) of 6 mL/kg predicted body weight (PBW) and plateau pressure (P_PLAT_) lower than 30 cmH_2_O has been shown to lead to improved outcomes [3–5]. This is likely explained by decreasing overdistention and alveolar wall stress, and consequently decreased local and systematic inflammatory response [6]. Recent data suggest that reducing driving pressure (the difference between P_PLAT_ and positive end-expiratory pressure (PEEP) (P_PLAT_-PEEP)), using MV with even lower V_T_, and limiting respiratory rate may offer additional benefit [7–11].The use of lower V_T_ and lower respiratory rate may be limited by decreased elimination of CO_2_ with resulting hypercapnia and respiratory acidosis. This may in turn lead to increased pulmonary shunt, elevated intracranial pressure, pulmonary hypertension, decreased myocardial contractility, decreased renal and splanchnic blood flow and the release of endogenous catecholamines [12, 13]. Moderate permissive hypercapnia is generally well tolerated when ventilating in a lung-protective manner. But the effect on survival of hypercapnic acidosis compared to low-tidal volume remains unclear [14]. In patients with normal kidney function, metabolic adaptation will compensate for respiratory acidosis, but this process takes some days.

In 1977 Kolobow et al. evaluated the use of extracorporeal CO_2_ removal (ECCO_2_R) in an animal model [15]; subsequently, Gattinoni et al. first used ECCO_2_R in patients with acute respiratory failure in 1986 [16]. ECCO_2_R can be performed using a blood flow (Qb) ranging from 300 to 1500 mL/min. Published experience with ECCO_2_R in ARDS patients consists mainly of cohort studies [17–21]. Some authors reported the use of ECCO_2_R in series with continuous renal replacement therapy (CRRT), offering an additional method of correcting acidosis. In these studies only patients who presented with acute kidney injury and ARDS were included [17–19].

Using a previous generation of pumpless arterio-venous ECCO_2_R, Bein et al. showed that this technique allowed protective MV with V_T_ of 3 mL/kg [22]. Fanelli et al. demonstrated that low-flow ECCO_2_R can safely be applied to facilitate ultra-protective MV with V_T_ of 4 mL/kg [23]. Since these effects were evaluated over several days, also metabolic adaption and improvement of gas exchange may have contributed to the pH, and the exact contribution of ECCO_2_R on pH control versus mechanical ventilation is uncertain.

The aim of our pilot study was to explore the short-term (2-h) effects of low-flow ECCO_2_R in steady state conditions in ARDS patients. In addition, we studied feasibility and adverse events of this treatment with special emphasis on bleeding and thrombosis.

## Methods

### Setting and patients

A prospective pilot study was conducted in the Intensive Care Unit of Ghent University Hospital, between December 2013 and May 2015. The ICU comprises a 14 beds medical, a 22 beds surgical and 10 beds cardiac surgical ICU. A convenience sample of 10 patients was included. Patients had to meet all of the following criteria for inclusion: 1. acute onset of moderate or severe ARDS, with a PaO_2_/FiO_2_ < 150 mmHg [24], 2. at least two hours of MV applying a V_T_ of 8 mL/kg PBW or less, with a positive end-expiratory pressure (PEEP) of 5 cmH_2_O or more, and FiO_2_ titrated to maintain an arterial oxygen saturation of 88–95% (PaO_2_ 55–80 mmHg), 3. P_PLAT_ of 28cmH_2_O or higher, 4. respiratory acidosis with pH < 7.25, 5. informed consent obtained from the patient or the proxy. Exclusion criteria were: 1. age < 18 years, 2. pregnancy, 3. obesity with a body mass index higher than 30 kg/m^2^, 4. contraindication for anticoagulation with unfractionated heparin, 5. Underlying chronic restrictive cause of respiratory acidosis, such as severe chest wall abnormalities.

Inhaled nitric oxide, muscle relaxants, and prone positioning were administered according to the discretion of the attending physician. PBW was calculated based upon patients’ length (measured with a measuring tape), for male patients: 50 + 0.91 · (cm of height – 152.4) kg, and for female patients: 45.5 + 0.91 · (cm of height – 152.4) kg [3]. Sedation was monitored at 8-h intervals, using the Richmond Agitation Sedation Score (RASS).

### Study design

This paper discusses the results of a prospective single center pilot study with a crossover design.

To assess the immediate effects of ECCO_2_R on gas exchange, we used a crossover design alternating periods with (“on”) and without (“off”) ECCO_2_R. The first 2-h study period without ECCO_2_R started as soon as the venous access for ECCO_2_R was in place. Following this, a first 2-h “on”-period was initiated with a Qb of 300 mL/min. The fraction of delivered oxygen via the oxygenator was kept at 1.0 with a gas flow of 7 L/min during “on”-periods. Hereafter followed the second 2-h “off”-period by setting the gas flow through the ECCO_2_R oxygenator at 0 L/min. Qb remained unchanged during this second “off”-period. During these 3 study periods, the settings for MV remained unchanged. After this second “off”-period, a second “on”- period was started, the gas flow was set again at 7 L/min and the Qb was increased to 400–500 mL/min. This “on”-period lasted as long as clinically indicated.

### Extracorporeal CO_2_ removal

Veno-venous ECCO_2_R was performed with the Abylcap® (Bellco®) device, mounted on a “Bellco” extracorporeal therapy machine. The Lynda® with a theoretical maximal Qb of 400 mL/min was used in the first 7 patients and the Amplya**™** with a theoretical maximal Qb of 500 mL/min in the others.

A dedicated team of intensivists, dialysis nurses, ICU nurses, and perfusionists managed the extracorporeal therapy.

The Abylcap® device contains the Lilliput 2 (LivaNova) oxygenator; this is a polymethylpentene hollow fiber oxygenator, phosphorylcholine coated, with a surface of 0.67m^2^ and a priming volume of 90 mL. The priming volume of the entire heparin-coated set without oxygenator is 109 mL. Venous access was obtained preferentially via the femoral vein with a 24 cm, 13.5 French double lumen catheter (Niagara**™**, Bard). Blood was pumped through the oxygenator via a non-occlusive roller pump.

Anticoagulation was performed with unfractionated heparin (UFH). After an initial bolus dose of 10 IU UFH/kg, heparin infusion was titrated to achieve an activated clotting time (ACT) of 180–220 s. ACT was measured every 30 to 60 min and once heparin titration was stable, every 4 h. ACT was monitored with the Medtronic ACT plus® system or the Hemochron ® signature elite.

### Mechanical ventilation

During the first six hours of the study period, the treating physicians were advised not to change the MV settings. This allowed evaluating the effects of ECCO_2_R alone during this initial study period. From the start of the second “on”-period, MV was left at the discretion of the treating physician; with the only recommendation that of ventilating the patient as lung-protective as possible. Practically, MV settings were aimed toward P_PLAT_ < 25cmH2O and V_T_ < 6 mL/PBW. The “higher FiO_2_ and lower PEEP” protocol of the ALVEOLI study was proposed [25]. We recommended setting of respiratory rate lower than 30/min. Inversed ratio was not allowed.

### Data management & analysis

During the first 6-h study period, MV parameters, arterial blood gas analysis and ECCO_2_R parameters were collected every two hours. Hereafter, data were recorded per 8 h. In addition, all adverse events, with special emphasis on bleeding events, thrombosis, and transfusion requirements were prospectively recorded.

The Murray score was calculated using the calculator on the “Cesar-trial” website http://cesar.lshtm.ac.uk/murrayscorecalculator.htm.

In order to have an objective judgment for bleeding, this was scored according the Bleeding Academic Research Consortium (BARC), and the Global Utilization of Streptokinase and Tpa for Occluded arteries (GUSTO) definitions for bleeding [26]. For a detailed description of the BARC and GUSTO definitions, please find an additional file as an online supplement (Additional file 1).

The primary endpoint was a reduction of 20% in arterial carbon dioxide pressure (PaCO_2_) after the two hours of ECCO_2_R therapy. Secondary endpoints were the short-term effect of ECCO_2_R on pH, feasibility of performing the technique, and bleeding and circuit thrombosis during ECCO_2_R treatment. In addition, we report the ventilation parameters and blood gasses after 5-d of ECCO_2_R.

A statistical package (SPSS Statistics version 22 (IBM®) was used to perform statistical analysis. Since the small number of patients included, data were considered not normally distributed. Variables were reported as median [inter quartile range] or number (proportion). Comparison of related variables was tested with the Wilcoxon Signed Rank test. A *p*-value of 0.05 was considered significant.

## Results

The baseline characteristics of the included patients are presented in Table 1. The median duration of ECCO_2_R therapy was 6 days [5; 12]. Four patients were initiated on CRRT during the study. CRRT was started at earliest after 10 h of ECCO_2_R therapy, so after the initial “off-on-off” evaluation. When CRRT was used, this was with separate vascular access. The maximum Qb during the second on-period of ECCO_2_R was 400 mL/min [399; 410]. At day 1 median heparin dosage was 19.5 IU/kg/h [11.0; 25.4] with an ACT of 211 s [186; 225].

**Table 1:** Baseline characteristics

Baseline characteristics	N (%) or median [IQR]
Number of patients included	10 (100%)
Age (y)	50.5 [34.8; 63.3]
Gender	Male 6 (60%)/female 4 (40%)
SAPS 3 score	72.5 [61.0; 79.3]
Characteristics at inclusion	
Length of ICU stay before inclusion (h)	26 [20.5; 67.5]
Days with ARDS criteria on CT scan or chest radiography on the day of inclusion	2 [1; 3.25]
SAPS 3 score	69.5 [57.75; 79.25]
Vasopressor use	10 (100%)
RRT use	0
PaCO2 (mmHg)	68.3 [57.7; 86.2]
pH	7.21 [7.11; 7.23]
PaO2/FiO2 (mmHg)	83 [67.6; 121.1]
Murray score	3.5 [3.2; 3.5]
Moderate ARDS (PaO_2_/FiO_2_: 100–200 mmHg)	4 (40%)
Severe ARDS (PaO_2_/FiO_2_ < 100 mmHg)	6 (60%)
Nitric Oxide inhalation	6 (60%)
Neuromuscular blockers	10 (100%)
Prone ventilation	2 (20%)
P_PLAT_ (cmH2O)	31.5 [28.8; 35.5]
Driving pressure (P_PLAT_ –PEEP) (cmH_2_O)	19.0 [17.5; 24.0]
V_T_/PBW (mL/kg)	6.9 [6.31; 7.48]

*y* Years, *SAPS 3* simplified acute physiology score 3, *RRT* Renal replacement therapy, *PaCO*_*2*_ Partial pressure of arterial carbon dioxide, *PaO*_*2*_ */FiO*_*2*_ the ration of partial pressure of arterial oxygen and fraction of inspired oxygen, *P*_*PLAT*_ Plateau pressure, *V*_*T*_ tidal volume, *PBW* Predicted body weight

ICU survival was 70%, hospital survival was 60%, and survival at day 28, and 90 was respectively 60% and 60%. In 4 patients, ECCO_2_R was the upper limit of life support, as after team discussions, these patients were not deemed to be candidates for extracorporeal membrane oxygenation (ECMO) therapy. Three patients died in the ICU because of limitations in therapy unrelated to ECCO_2_R.

### Short term effects of ECCO_2_R

**Fig. 1:**
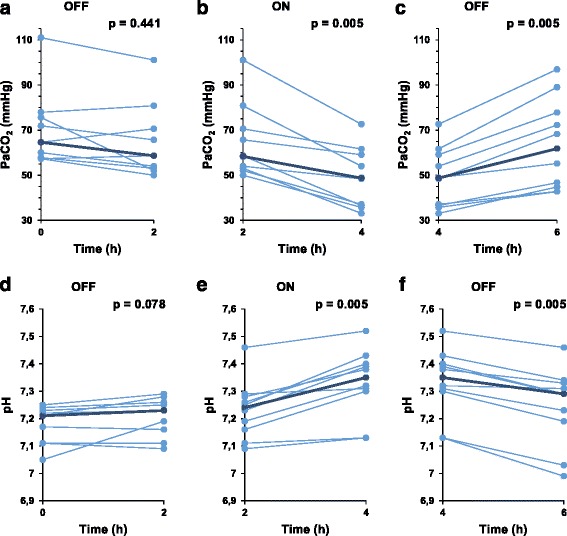
Evolution of PaCO2 during the 6-h off-on-off period. **a**: Evolution of PaCO2 during the first 2-h off period. **b**: Evolution of PaCO2 during the first 2-h on period. **c**: Evolution of PaCO2 during the second 2-h off period. **d**: Evolution of pH during the first 2-h off period. **e**: Evolution of pH during the first 2-h on period. **f**: Evolution of pH during the second 2-h off period. This figure was created with Excel (Office)

The protocol required settings of the MV to be unaltered during the 6-h crossover, in order to be able to study the short-term effects of ECCO_2_R in steady state conditions. In one patient the treating physician modified MV settings during the first off period, with resulting decrease of PaCO_2_ (132 to 58 mmHg). In order to secure the detection of the effect of ECCO_2_R, ventilation conditions remained unchanged for the next four hours. PCO_2_ values of this patient have been withdrawn from analysis of the first “off” -period (fig. 1a). Median PaCO_2_ remained stable during the first 2-h “off”-period (delta change −8.6% [−11.4; 3.0], *p* = 0.441), decreased during the “on”-period (−28.4% [−12.2; −34.3], *p* = 0.005), and subsequently increased during the second “off”-period (32.6% [18.5; 36.8], p = 0.005). During the 2-h on period 6 patients (60%) had a decrease in PaCO_2_ of 20% or greater (fig. 1b).

Median pH remained stable during the first 2-h “off”-period (delta change 0.28% [−0.07; 0.76], *p* = 0.078) (figure1d), increased during the first “on”-period (1.59% [0.49; 2.02], p = 0.005) (fig. 1e), and decreased again during the second “off”-period (−1.28% [−1.50; −0.77], p = 0.005) (fig. 1f).

### Feasibility and adverse events

Insertion of the double lumen catheter was troublesome in 2 patients (20%). One patient (10%) had transfusion of 1 unit of RBCs after insertion of the catheter. One patient (10%) had premature cessation after 6-h of ECCO_2_R for presumed hemorrhagic pericardial effusion. This finding was not confirmed after cessation of therapy.

During ECCO_2_R, bleeding was observed in 5 patients (Table 2). Patient 3 had a nosebleed and was bleeding at the insertion points of the ECCO_2_R catheter and the central venous catheter. Patient 4 was bleeding at the insertion point of the ECCO_2_R catheter, had a pharyngeal bleeding and hematuria. Patient 5 was bleeding during insertion of the catheter. Patient 7 had minor bleeding during tracheal and pharyngeal aspirations. Patient 9 was bleeding at the insertion point of the ECCO_2_R catheter. According to the GUSTO criteria and BARC definitions, bleeding was scored in 4 patients (40%) as moderate or 3a, and in 1 patient (10%) as mild or 2 [26]. Five patients (50%) received transfusion of red blood cells. In none of the patients, bleeding resulted in hemodynamic instability.

In 3 patients (30%), clots were observed in the circuit. In 2 patients (20%), clotting of the circuit occurred after temporary stop of the heparin infusion because of increased ACT results.

We observed transient and short-lasting alkalemia (defined as pH > 7.45) in 8 patients (80%).

**Table 2:** Bleeding, clotting in circuit and transfusion needs

Patient no	Clotting in circuit	Bleeding	Transfusion	Cumulative Units of PC during ECCO_2_R	GUSTO bleeding criteria	BARC type
1				0		0
2				0		0
3		Y	Y	4	moderate	3a
4	Y	Y	Y	4	moderate	3a
5	Y	Y	Y	3	moderate	3a
6				1		0
7		Y		0	mild	2
8				1		0
9		Y	Y	3	moderate	3a
10	Y		Y	2		0
total	3/10	5/10	5/10	1.5 [0; 3.25]		

“*Y*” yes, an empty box means no, *PC* Packed Cells, *GUSTO* Global Utilization of Streptokinase and Tpa for Occluded arteries definition of bleeding, *BARC* Bleeding Academic Research Consortium definition for bleeding

### Evolution of ventilation and blood gasses after 5-d ECCO_2_R

Plateau pressure, P_PLAT_-PEEP and V_T_/PBW were significantly reduced after 5 days of therapy (Table 3). This was accompanied with a steady increase of bicarbonate and pH. Median pH at the moment of decision of weaning from ECCO_2_R was 7.42 [7.39; 7.44].

A significant increase in PaO_2_/FiO_2_ was noted, indicating amelioration of ARDS.

**Table 3:** Evolution in ventilatory measurements

	Inclusion	Day 2	Day 3	Day4	Day 5	Difference	% change	p
P_PLAT_ (cmH_2_O)	31.5 [28.8; 35.5]	25.0 [22.5; 30.5]	26.0 [22.0; 29.5]	25.0 [22.0; 27.0]	24.0 [21.0; 26.0]	10.0 [4.5; 12.5]	−31.4 [−36.2; −15.4]	0.008
V_T_ (mL)	334 [317; 469]	300 [270; 353]	318 [270; 390]	300 [251; 344]	308 [243; 347]	69.0 [20.0; 90.0]	−20.9 [−26.3; −5.17]	0.008
V_T_/PBW (mL/kg)	6.85 [6.31; 7.48]	5.60 [4.90; 6.05]	5.80 [4.75; 6.45]	5.30 [4.55; 6.20]	5.00 [4.55; 5.95]	1.6 [0.8; 2.15]	−23.7 [−31.3; −13.9]	0.008
Driving Pressure: P_PLAT_ –PEEP (cmH_2_O)	19.0 [17.5; 24.0]	14.0 [11.0; 18.5]	15.0 [11.0; 19.0]	15.0 [11.0; 17.0]	14.0 [10.0; 16.5]	9.0 [3.5; 12]	−39.1 [−52.2; −21.7]	0.012
PEEP (cmH_2_O)	12.0 [9.0; 12.75]	11.0 [8.5; 14.5]	11.0 [8.0; 13,5]	10.0 [8.0; 11.50]	10.0 [8.0; 10.0]	2.0 [−1.0; 2.0]	16.7 [−16.7; 16.7]	0.305
Respiratory Rate	32.0 [27.8; 33.0]	30.0 [27.5; 31.5]	30.0 [27.0; 31.5]	30.0 [27.0; 33.5]	30.0 [26.0; 33.5]	3.0 [−1.0; 6.0]	9.1 [−3.0; 20.0]	0.235
pH	7.21 [7.11; 7.23]	7.33 [7.29; 7.38]	7.38 [7.31; 7.42]	7.39 [7.34; 7.44]	7.43 [7.40; 7.44]	0.23 [0.20; 0.26]	3.19 [2.77; 3.63]	0.008
PaO_2_/FiO_2_ (mmHg)	83.0 [67.6; 105.4]	131.4 [90.2; 212.7]	137.2 [118.9; 223.8]	142.0 [116.1; 245.5]	233.6 [135.7; 320.1]	91.9 [29.9; 213.8]	144.0 [30.8; 232.2]	0.011
PaCO_2_ (mmHg)	68.3 [57.3; 86.2]	50.3 [42.9; 63.9]	51.5 [48.2; 62.2]	56.8 [46.6; 63.1]	59.3 [48.2; 60.9]	14.6 [4.0; 35.8]	22.2 [6.9; 38.0]	0.021
HCO3^−^	22.6 [22.1; 29.6]	25.7 [20.3; 34.1]	25.7 [25.3; 30.0]	29.1 [27.9; 39.3]	35.3 [29.4; 41.9]	−7.6 [−12.1; −3.2]	−34.2 [−51.2; −14.8]	0.021
FiO_2_	0.83 [0.68; 1.00]	0.55 [0.40; 0.75	0.55 [0.35; 0.73]	0.60 [0.35; 0.73]	0.50 [0.35; 0.73]	0.25 [0.05; 0.5]	45.5 [5.9; 50.0]	0.017

Data are expressed as median [IQ25; IQ75]. Difference: the median of the difference between the data at time of inclusion and at day 5

## Discussion

In this prospective crossover pilot study in patients with moderate or severe ARDS, we found that with stable MV settings, low flow ECCO_2_R resulted in a rapid decrease of PaCO_2_ with almost one-third. During the whole treatment period, we observed a high incidence of bleeding and thrombosis.

In our study, we evaluated the short-term effects of ECCO_2_R in steady state MV conditions, using study periods of 2-h. It is therefore reasonable to presume that changes in PaCO_2_ can be attributed to the effects of ECCO_2_R only, and were not obscured by an improvement of respiratory function, metabolic compensation, or change in settings of the mechanical ventilator. This is an important aspect that was not studied before. Several other studies have reported the beneficial effects of ECCO_2_R on PaCO_2_ in ARDS patients. However these differed from our study in study design, the non-reporting of short-term effects, combined use with CRRT, and the use of other devices such as pumpless arterio-venous devices [17, 19, 21–23, 27]. An important advantage of this low flow veno-venous ECCO_2_R treatment is that Qb up to 500 mL/min can be achieved by using a catheter that is also used for CRRT, making it a less invasive technique compared with others that are using an arterial catheter or large bore wire-reinforced ECMO cannulas [22, 23, 27–30]. However, it should be noted that despite our use of relatively small-bore catheters of 13.5F, catheter insertion was difficult in two patients.

We observed some variability in response to ECCO_2_R. A possible explanation for this may be a difference in volume of distribution of CO_2_. Two main components that determine CO_2_ removal are Qb and sweep gas flow. Here we used a Qb of 300 mL/min during the first “on”- period of our study, in which we demonstrated an almost 30% decrease of CO2. In the second “on”-period, a median Qb of 400 mL/min allowed adequate removal of CO2. Our findings are in contrast to others who have reported higher Qb. For instance, Karagiannidis et al. suggested in their study in pigs a Q_B_ of 750 to 1000 mL/min for efficient CO2 removal [31], and Bein et al. used in vivo a Qb of 2.2 L/min for the A-V iLA device [27, 32]. An explanation for the efficacious CO_2_ removal despite low Qb in our study may be the deep sedation used in our patients, limiting CO_2_ production. Another determinant for CO_2_ removal is sweep gas flow. In this study the sweep gas flow was kept at 7 L/min, with a FiO_2_ of 1.0, since we found in a previous study that CO_2_ removal rate only marginally improved above gas flows of 6 L/min [33].

After 5 days of ECCO_2_R we observed a marked decrease in P_PLAT_, P_PLAT_-PEEP and V_T_. This resulted in a more lung protective strategy of MV with ventilation settings of V_T_, P_PLAT_ and driving pressure below the currently recommended [7–10]. Decreased V_T_, P_PLAT_ and P_PLAT_-PEEP without an increase in PaCO_2_ may offer benefits for ARDS patients, since it may lead to a further decrease of inflammatory mediators in broncho-alveolar fluid and systemic circulation, which in turn may contribute to less lung damage and improved outcomes [17, 23]. Since pH was still 7.43 at day 5, it might have been feasible to achieve even more pronounced decreases of mechanical ventilation settings.

Adequate anticoagulation remains challenging in the management of ECCO_2_R-patients. In our study, 30% of all patients experienced some form of circuit thrombosis. This was accompanied with a temporal increase in PaCO_2_ and consequent change of the ventilator to less lung protective settings. We also observed bleeding and need for transfusion of red blood cells in 50% of patients. Others have also reported bleeding and blood loss as an important complication of ECCO_2_R [22, 27, 34]. This complication seems to occur more frequently in ECCO_2_R compared to CRRT (16%), a well-established extracorporeal therapy for AKI [35]. We can only speculate on the etiology of bleeding in these patients. Potential causes could be the poor correlation of both ACT and aPTT with the heparin concentration [36, 37], and the activation of platelets and induction of other coagulation abnormalities by unfractionated heparin and/or the ECCO_2_R circuit [38].The use of visco-elastic monitoring such as TEG, RoTEM or Sonoclot, preferable in combination with a platelet function test may allow better monitoring of coagulation in these patients.

Strengths of our study are the prospective crossover design with detailed short-term evaluation of the effects of ECCO_2_R. This allowed us to evaluate the effects of ECCO_2_R with the patient serving as its own control. In addition, we reported the adverse events, more in particular bleeding, meticulously and in a structured way according to the validated GUSTO and BARC scales. As until now, no RCT’s have proven advantage over permissive hypercapnia, it is important to quote the principle ‘primum non nocere’ to guide clinicians considering ECCO_2_R. Finally, this study evaluated ECCO_2_R in moderate (*n* = 4) and severe ARDS patients (*n* = 6), and as such reflects the recommended cohort of ARDS patients that may benefit of ECCO_2_R [24]. We want to discuss the following limitations of this pilot study. We report data on a small number of patients in a single center setting. Second, we may have underestimated the short-term effects of ECCO_2_R during the first six hours of our study period as we only used a Qb of 300 mL/min. Third, reduction of V_T_, P_PLAT_ and driving pressure were the main goals of management of MV in this study. Recently, the large epidemiologic LUNG SAFE study showed that a lower respiratory rate is also associated with better patient outcomes [39]. These results were not available at time of the initiation of the study, and decreasing respiratory rate was therefore not set as a goal for MV here. Finally, we can only speculate on the efficacy of ECCO_2_R using low Qb in patients who are more awake**.**

## Conclusions

We showed the feasibility of CO2 removal with a low flow ECCO_2_R device in patients with moderate and severe ARDS. Crossover design allowed us to evaluate the short-term effects of ECCO_2_R in steady state conditions, with the patient serving as his own control, and demonstrated an almost one-third decrease of PaCO_2_ within a 2-h study period. However, ECCO_2_R treatment was associated with bleeding in half of the patients and circuit thrombosis in one third. These complications should be explored in greater detail and may be a major barrier for larger studies on efficacy of ECCO_2_R.

## Additional file

Additional file 1: In this Word file the “Bleeding Academic Research Consortium Definition for Bleeding” and the “Global Utilization of Streptokinase and Tpa for Occluded arteries definition of bleeding” are described. (DOC 36 kb)

## Abbreviations

ACT: activated clotting time
AE: adverse events
aPTT: activated partial tromboplastin time
ARDS: acute respiratory distress syndrome
BARC: Bleeding Academic Research Consortium
COPD: chronic obstructive pulmonary disease
CRRT: continuous renal replacement therapy
ECCO_2_R: extracorporeal CO_2_ removal
GUSTO: Global Utilization of Streptokinase and Tpa for Occluded arteries
MV: mechanical ventilation
PaCO_2_: arterial carbon dioxide pressure
PaO_2_/ FiO_2_: the ratio of the arterial oxygen partial pressure to fractional inspired oxygen
PBW: predicted body weight
PC: packed cells
PEEP: positive end-expiratory pressure
P_PLAT_: plateau pressure
Qb: blood flow
RBC: Red blood cells
RRT: renal replacement therapy
SAPS 3: simplified acute physiology score 3
V_T_: tidal volume
