# Supplementary material for: Low flow extracorporeal CO2 removal in ARDS patients: a prospective short-term crossover pilot study
Source: BMC Anesthesiol. 2017 Nov 28;17:155. doi: 10.1186/s12871-017-0445-9 (PMC5704518; doi:10.1186/s12871-017-0445-9)
Supplement: Additional file 1: — In this Word file the “Bleeding Academic Research Consortium Definition for Bleeding” and the “Global Utilization of Streptokinase and Tpa for Occluded arteries definition of bleeding” are described. (DOC 36 kb) [file 12871_2017_445_MOESM1_ESM.doc]

**Bleeding Academic Research Consortium Definition for Bleeding (BARC)**

Type 0: no bleeding

Type 1: bleeding that is not actionable and does not cause the patient to seek unscheduled performance of studies, hospitalization, or treatment by a healthcare professional; may include episodes leading to self-discontinuation of medical therapy by the patient without consulting a healthcare professional

Type 2: any overt, actionable sign of haemorrhage (eg, more bleeding than would be expected for a clinical circumstance, including bleeding found by imaging alone) that does not fit the criteria for type 3, 4 or 5 but does meet at least one of the following criteria: (1) requiring nonsurgical medical intervention by a healthcare professional, (2) leading to hospitalization or increased level of care, or (3) prompting evaluation

Type 3:

Type 3a

- Overt bleeding plus haemoglobin drop of 3 to <5g/dL * (provided haemoglobin drop is related to bleed)
- Any transfusion with overt bleeding

Type 3b

- Overt bleeding plus haemoglobin drop ≥5g/dL* (provided haemoglobin drop is related to bleed)
- Cardiac tamponnade
- Bleeding requiring surgical intervention for control (excluding dental/nasal/skin/haemorrhoid)
- Bleeding requiring intravenous vasoactive agents

Type 3c

- Intracranial haemorrhage (does not include microbleeds or haemorrhagic transformation, does include intraspinal)
- Subcategories confirmed by autopsy or imaging or lumbar puncture
- Intraocular bleed compromising vision

Type 4: CABG-related bleeding

Perioperative intracranial bleeding within 48h

Reoperation after closure of sternotomy for the purpose of controlling bleeding

Transfusion of ≥5 U whole blood or packed red blood cells within a 48h period**

Chest tube output ≥2L within a 24h period

Type 5: fatal bleeding

Type 5a

- Probable fatal bleeding; no autopsy or imaging confirmation but clinically suspicious

Type 5b

- Definite fatal bleeding; overt bleeding or autopsy or imaging confirmation

CABG: coronary artery bypass graft. Platelet transfusions should be recorded and reported but are not included in these definitions until further information is obtained about the relationship to outcomes. If a CABG-related bleed is not adjudicated as at least a type 3 severity event, it will be classified as a not a bleeding event. If a bleeding event occurs with a clear temporal relationship to CABG (ie, within a 48h time frame) but does not meet type 4 severity criteria, it will be classified as not a bleeding event.

*Corrected for transfusion (1U packed red blood cells or 1 U whole blood = 1g/dL haemoglobin)

**Cell saver products are not counted.

**Global Utilization of Streptokinase and Tpa for Occluded arteries definition of bleeding**

Severe or life-threatening

- Intracerebral haemorrhage
- Resulting in substantial hemodynamic compromise requiring treatment

Moderate

- Requiring blood transfusion but not resulting in hemodynamic compromise

Mild

- Bleeding that does not meet above criteria

Reference: 30. Mehran R, Rao SV, Bhatt DL, Gibson CM, Caixeta A, Eikelboom J, Kaul S, Wiviott SD, Menon V, Nikolsky E, Serebruany V, Valgimigli M, Vranckx P, Taggart D, Sabik JF, Cutlip DE, Krucoff MW, Ohman EM, Steg PG, White H: **Standardized Bleeding Definitions for Cardiovascular Clinical Trials: A Consensus Report From the Bleeding Academic Research Consortium**. *Circulation* 2011, **123**:2736–2747.
